# Solar‐Driven Rechargeable Lithium–Sulfur Battery

**DOI:** 10.1002/advs.201900620

**Published:** 2019-05-24

**Authors:** Peng Chen, Guo‐Ran Li, Tian‐Tian Li, Xue‐Ping Gao

**Affiliations:** ^1^ Institute of New Energy Material Chemistry School of Materials Science and Engineering Renewable Energy Conversion and Storage Center Nankai University Tianjin 300350 China

**Keywords:** carbon electrodes, lithium–sulfur batteries, perovskite solar cells, solar rechargeable batteries

## Abstract

Solar cells and rechargeable batteries are two key technologies for energy conversion and storage in modern society. Here, an integrated solar‐driven rechargeable lithium–sulfur battery system using a joint carbon electrode in one structure unit is proposed. Specifically, three perovskite solar cells are assembled serially in a single substrate to photocharge a high energy lithium–sulfur (Li–S) battery, accompanied by direct conversion of the solar energy to chemical energy. In the subsequent discharge process, the chemical energy stored in the Li–S battery is further converted to electrical energy. Therefore, the newly designed battery is capable of achieving solar‐to‐chemical energy conversion under solar‐driven conditions, and subsequently delivering electrical energy from the stored chemical energy. With an optimized structure design, a high overall energy conversion efficiency of 5.14% is realized for the integrated battery. Moreover, owing to the self‐adjusting photocharge advantage, the battery system can retain high specific capacity up to 762.4 mAh g^−1^ under a high photocharge rate within 30 min, showing an effective photocharging feature.

Pollutants released by the abusing of fossil fuels have gained great attentions in recent years, and exploring an alternative clean and renewable energy is one of the most appealing solutions. Sunlight is the source of almost all the energy on the earth, which is totally free, highly available, and environmental benign. More importantly, sunlight radiation could be easily converted into electricity via photovoltaic technology. Since electric energy is the foundation of modern industry and society, the solar to electric conversion is of great value for clean and renewable energy application. However, the intermittent nature of sunlight leads to unsteady output of solar cells, and makes it undesirable for direct and sustainable energy utilization. Secondary batteries are widely used to store electric energy as chemical energy, and convert chemical energy directly to electrical energy when needed. Therefore, developing efficient and cheap technologies to integrate solar cells with secondary batteries is a viable solution for the desirable application of solar energy.

In a typical natural process, the plants can store the solar energy in carbohydrates by photosynthesis. Inspired by the plants, researchers have developed several solar storable battery modes to convert solar energy to chemical energy and thus realizing the storage of solar energy. Among these modes, external connected mode (ECM) is widely used for commercial solar energy storage system, which usually consists of individual Si‐based solar cells and secondary batteries (Li‐ion batteries^1^, ^2^, ^3^, ^4^, ^5^, ^6^ or redox flow batteries^7^, ^8^, ^9^, ^10^, ^11^, ^12^). By using external circuit to mechanically connect the two commercial devices together, ECM could achieve relatively high overall photoconversion efficiency (PCE) and large‐scale energy storage. However, the additional electric elements, like direct current‐direct current (DC–DC) converter and battery management system, would lead to lower integration level and higher cost. In recent years, sharing electrolyte mode (SEM) is proposed as a new concept for building an integrated solar storage system. In SEM, both solar cells and secondary battery are integrated into one structure unit with sharing electrolyte to achieve in situ solar–electric–chemical energy conversion and storage. Herein, the key issue is the transportation of the photogenerated electrons from the solar cell side to the energy storage electrodes, which are usually metal oxides,^13^ hydrogen storage materials,^14^ carbon materials,^15^ and photocatalysts^16^, ^17^, ^18^, ^19^, ^20^, ^21^, ^22^, ^23^ in different liquid electrolytes. It should be noted that the research of the solar storable battery based on SEM, with a relatively low overall efficiency (mostly less than 1%), is still in its infant stage. In particular, the major issue for the low efficiency is the unsatisfactory energy storage electrode materials, which are limited by the low working voltage provided by solar cells. Actually, in one structure unit with sharing electrolyte, it is impossible to further enhance the working voltage of the solar storable batteries driven by solar cells. Especially by DSSCs with liquid electrolyte, in which the series structure is unsuitable. To increase the working voltage, a solar storable battery should be designed on the basis of solid‐state solar cells, which are easily fabricated as a serially connected solar cell pack in one unit. Additionally, such integrated solar storable battery should be effectively manipulated under smart control mode, especially for the photocharging process.

Recently, great progresses on highly performance solid‐state perovskite solar cells (PSCs) have been achieved,^24^, ^25^, ^26^, ^27^, ^28^, ^29^, ^30^, ^31^, ^32^, ^33^, ^34^, ^35^, ^36^, ^37^, ^38^, ^39^, ^40^, ^41^, ^42^, ^43^ making it possible to utilize those efficient all solid and high‐voltage photovoltaics for photocharging under continuous illumination. When fabricated on a specially etched FTO substrate, a serially connected perovskite solar cell pack can provide a high open‐circuit voltage up to 2.8 V with a maximum power point (MPP) near 2.4 V. Coincidently, high‐energy lithium–sulfur (Li–S) battery has a suitable charge voltage plateau near 2.4 V and cut‐off charge voltage at 2.8 V,^44^ which is perfectly appropriate for cobuilding PSCs based solar storable batteries as requested by voltage matching principle. Herein, we propose a joint electrode mode (JEM) for solar‐driven high‐energy Li–S battery by solid‐state PSCs. Typically, by using a stable carbon layer as joint electrode, we successfully integrate Li–S battery on PSCs for the first time with JEM design. Moreover, differently from conventional galvanostatic or constant‐ voltage charge mode in secondary batteries, the photocurrent is smartly self‐adjusted with the voltage increasing during photocharging process, leading to more sufficient redox reaction. Consequently, a high overall efficiency of 5.14% and high specific capacity up to 762.4 mAh g^−1^ under ultrafast photocharge rate of 2 C can be achieved. These results indicate that the JEM designed solar storable system in this work is of great promise for potential applications of solar energy in energy storage power station.

**Figure** **1** schematically shows the structure configuration and working mechanism of the integrated solar‐driven rechargeable Li–S battery. In the JEM, the sulfur‐based electrode is connected to the carbon layer as joint electrode of the three PSC units as shown in Figure S1 of the Supporting Information. During the photocharging process, perovskite absorber captures the photons to generate pairs of electrons and holes. The photogenerated electrons are injected rapidly into the conduction band of TiO_2_, and transferred further to the lithium metal electrode by the external circuit. Lithium ions in the electrolyte would be reduced to lithium metal by the photogenerated electrons immediately. Meanwhile, holes can be collected by the dense carbon layer, and then delivered onto the porous carbon layer. Herein, holes can oxidize polysulfide anions into the elemental sulfur on porous carbon layer. In such process, the solar energy can be directly converted to the chemical energy, and stored in Li–S battery. In the subsequent discharge process, the chemical energy stored in Li–S battery is released as the electrical energy by the conventional redox reaction. Specifically, lithium metal is oxidized to Li ions accompanying with releasing electrons. In the carbon electrode, sulfur is reduced into polysulfide anions after receiving electrons from Li anode. Therefore, the battery can achieve solar‐to‐chemical energy conversion and storage under solar‐driven condition, and subsequent chemical‐to‐electrical energy application.

**Figure 1:**
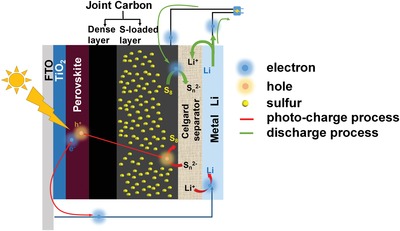
Schematic diagram of the fabricated PSC–Li–S battery.

To verify the working feasibility of the solar rechargeable battery, cyclic voltammograms (CVs) at a scan rate of 0.1 mV s^−1^ with the potential range of 1.7–2.8 V (vs Li/Li^+^) in Li–S cell are measured (**Figure** **2**a). There are two typical cathodic peaks, associated with the formation of soluble polysulfides (S*_n_* ^2−^, 4 ≤ *n* ≤ 8) and insoluble products (Li_2_S_2_/Li_2_S). Accordingly, in the subsequent anodic scan, two oxidation peaks are observed at 2.37 and 2.41 V (vs Li/Li^+^), corresponding to the oxidation of insoluble products (Li_2_S_2_/Li_2_S) to soluble polysulfides, and polysulfides to sulfur element. In this case, 2.37 V should be the minimum photocharging voltage provided by PSCs. To improve the overall efficiency,^45^ 2.41 V should also be the MPP of the solar cells unit. Figure 2b shows typical current density–voltage (*J*–*V)* characteristics of a single PSC. For the optimized carbon based single PSC, a short‐circuit photocurrent density of 23.1 mA cm^−2^, open‐circuit voltage (OCV) of 0.983 V, fill factor (FF) of 0.701, and PCE of 15.90% are obtained. To the best of our knowledge, our single device with PCE of 15.9% is one of the best reported carbon‐based PSCs.^46^, ^47^, ^48^, ^49^, ^50^, ^51^ To ensure a sufficient operating voltage for directly solar‐driving Li–S battery, at least three PSC units should be connected in series. In view of adjusting the MPP to around 2.4 V, we then design a serial connected structure of three PSCs on an etched FTO substrate. Photocharging process could be regulated by editing the photocurrent. Masks are used to control the active areas of PSCs, thus tuning the photocurrent. For the connected PSC unit with different active areas of 0.21, 0.42, and 0.63 cm^2^, we obtain the OCV of 2.79, 2.84, and 2.80 V, and PCE of 12.38%, 11.06%, and 11.34%, respectively (Figure 2c). Such OCV of PSCs is high enough to charge Li–S battery, especially for the connected PSC unit with the active area of 0.42 cm^2^. Furthermore, to investigate the photocurrent changing tendency in operating voltage range, a slow scan test is made in the voltage range of 2.4 to 2.7 V at a rate of 0.1 mV s^−1^. As shown in Figure 2d, the photocurrent would decrease quickly with the voltage increasing, showing a smart self‐adjusting feature and manipulating mode for photocharging process stem from the diode characteristics. In addition, to provide the working stability of the solar rechargeable battery, the PSC part would endure long time illumination in photocharging process. As shown in Figure 2e, both the single and connected PSCs present a steady power output feature during short‐time irradiation (300 s). However, during long‐time irradiation test, the performance of the device is slightly increased in the initial 15 min, and dropped from more than 14% to about 12% after 90 min (Figure S2, Supporting Information). Moreover, in a thermal stability test at 100 °C for 60 min, the devices show no degradation in performance (Figure S3, Supporting Information). Therefore, all the features of the connected PSC unit can ensure the working feasibility of the solar rechargeable battery, based on the voltage matching, working stability, and smart manipulating mode.

**Figure 2:**
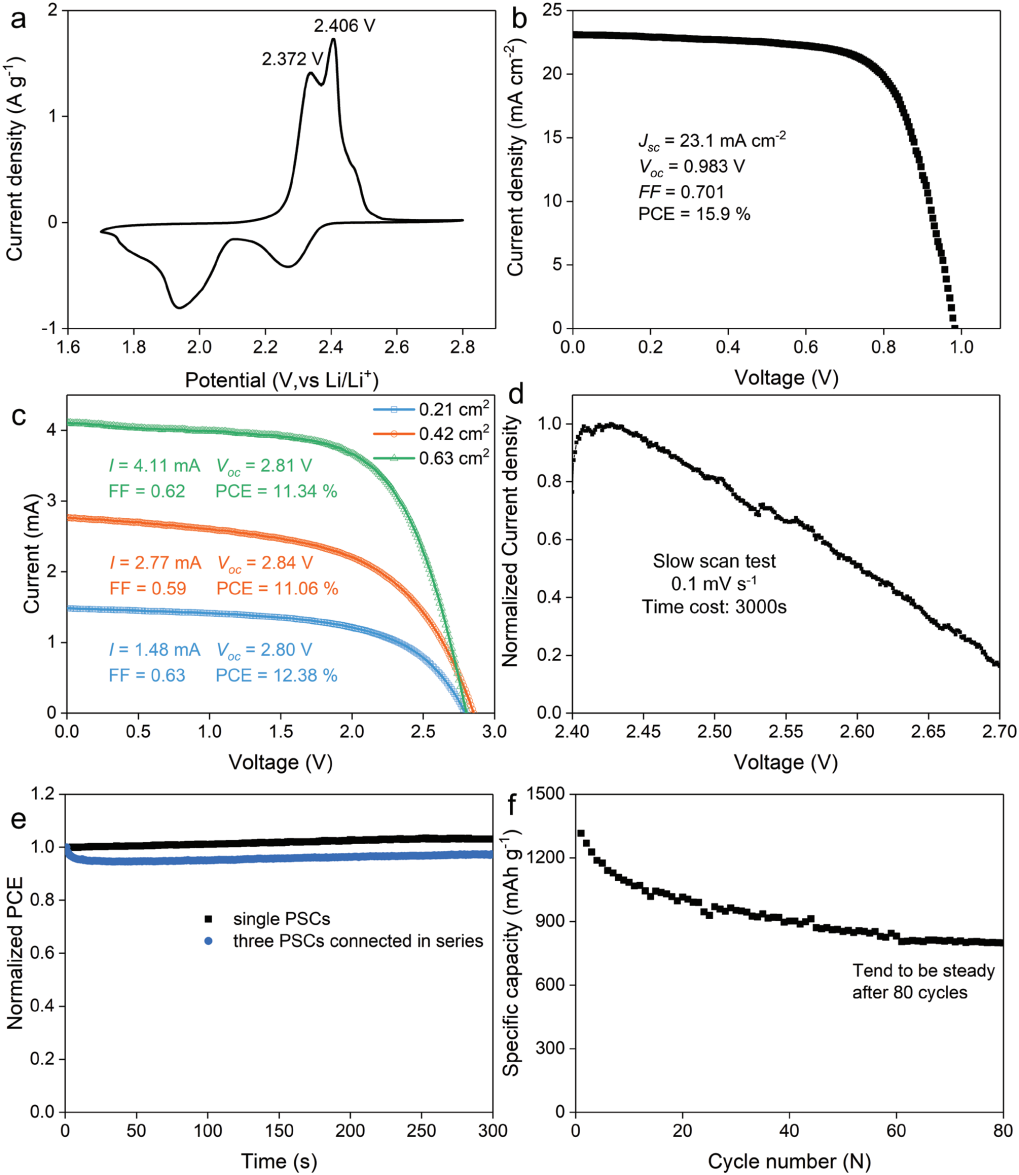
a) Cyclic voltammogram (CV) at a scan rate of 0.1 mV s^−1^ with the potential range of 1.7–2.8 V (vs Li/Li^+^) in Li–S cell. b) *J*–*V* curve for single PSC. c) *J*–*V* curves for the connected PSCs unit with different active area (the unit is fabricated by three single PSCs connected in series). d) Normalized photocurrent changing tendency in operating voltage range of 2.4–2.7 V. e) Stabilized power output measured at a bias voltage near the maximum power point for single and connected PSCs. f) Discharge capacity in the initial 80 cycles of a typical Li–S battery at 0.2 C rate.

To demonstrate the photocharging and electrochemically discharging characteristics, the integrated solar rechargeable battery is first cyclic charged and discharged using an automatic battery tester system (Land, China) at power supply mode. The current density is set as 0.2 C rate (1 C = 1675 mA g^−1^ for sulfur cathode), with the voltage range from 1.7 to 2.7 V. After 80 cycles by power supply mode, the capacity performance tends to be stable (Figure 2f), and the device is then tested at photocharge mode. Specifically, the battery is photocharged until the voltage reach 2.7 V, and galvanostatically discharged to 1.7 V at 0.2 C rate in the dark. When the active area of PSCs is determined as 0.42 cm^2^, the charge–discharge profiles for the last three cycles at power supply mode, and two cycles at photocharge mode are shown typically in **Figure** **3**a. Clearly, the discharge curves at both photocharge and power supply modes are almost identical to that in typical Li–S battery. However, it is of interest to note that the charge curve is remarkably different under photocharging mode. A sharp starting voltage peak, short voltage plateau, and a gradually smooth “voltage tail” are observed under photocharging, indicating a novel manipulating mode. It is like an initial fast‐charging process, which is then automatically switched to constant voltage process near the end of photocharging. Moreover, the discharging capacity could maintain at a same level as compared to power supply mode (Figure 3b). High rate tests are presented to make the comparison fairly. As shown in Figure S5 of the Supporting Information, while the charge time of 1C rate at power supply mode is similar to that at photocharge mode, however, the battery delivers the larger discharge capacity at photocharge mode (750 vs 600 mAh g^−1^). Therefore, the photocharge mode is more effective for charging Li–S battery.

**Figure 3:**
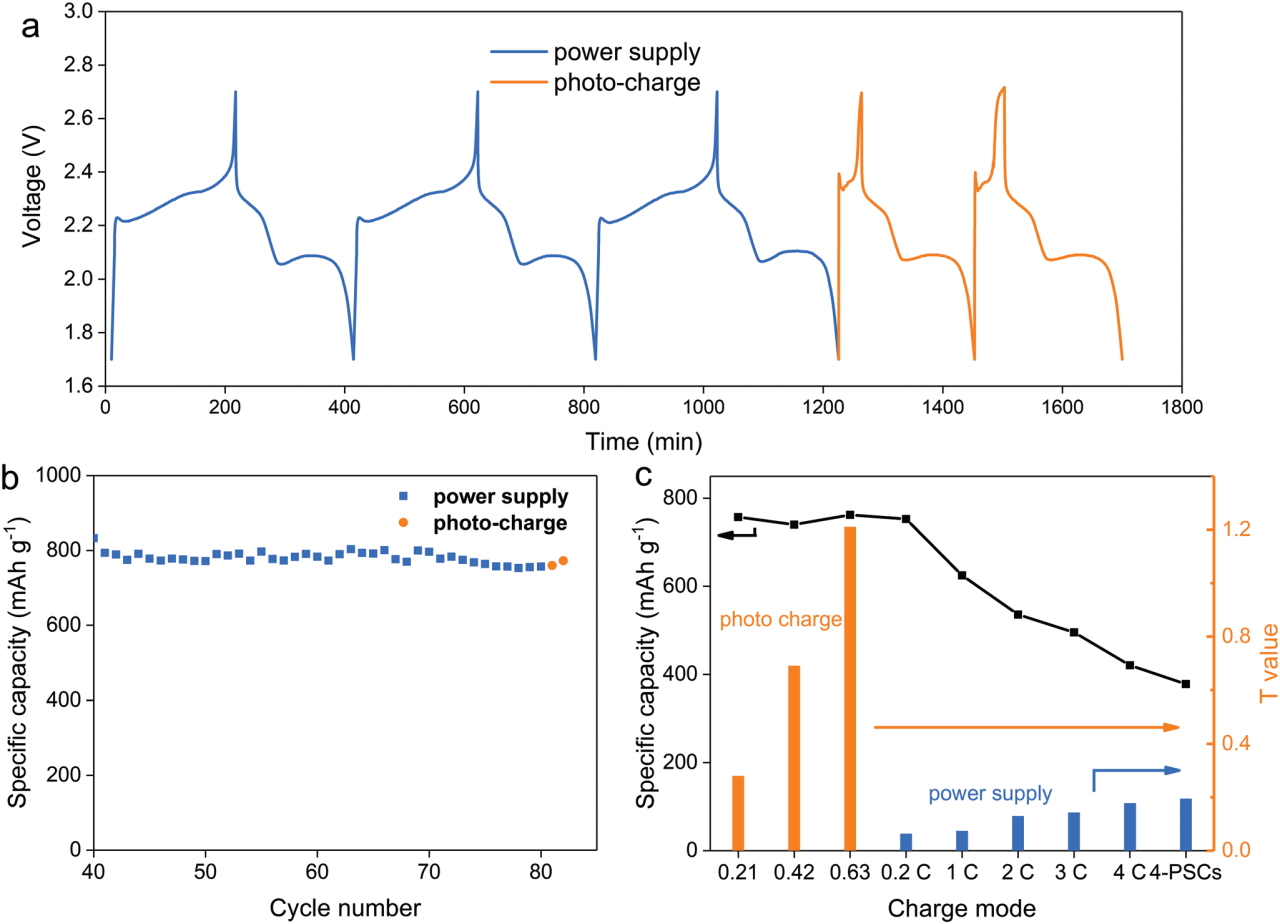
a) Voltage–time (*V*–*t*) curves of the last three cycles of power supply and two cycles of photocharge process (blue lines for power‐supply charge and discharge process at 0.2 C rate galvanostatically. Yellow lines for photocharge process using PSCs of 0.42 cm^2^ active area and galvanostatically discharged at 0.2 C rate). b) Cycle test results of discharge capacities for last 40 power‐supply cycles (blue) and 2 photocharge cycles (yellow). c) Discharge capacities (black line) and T values (blue and yellow columns) of the battery under different charge modes.

Generally, the charge efficiency of Li‐ion batteries at high rates is poor in a galvanostatic charge process. Especially, the time cost is poor in high voltage period due to the slow electrode kinetics. To improve the charge efficiency, dual‐mode is usually used for Li‐ion batteries, in which the constant voltage charging is continued in high voltage period right after the galvanostatic charge process. In order to evaluate the photocharge effect precisely, we introduce T factor here. Herein, T refers to the ratio of charge time at high‐voltage range to the charge time at low‐voltage range. Typically in a dual‐mode for Li‐ion batteries, a large current is initially used in the galvanostatic process to achieve fast charge rate, which is converted to a constant voltage process at high voltage region to fully charge the battery. In consequence, the dual‐mode would lead to large T factor. In Li–S battery, the boundary of high/low voltage is confirmed from the CVs test, the high‐voltage range is set at 2.4–2.7 V (Figure 2a). For example, when Li–S battery is charged at 0.2 C rate under power supply mode, it takes 187 min to elevate the voltage from 1.7 to 2.4 V, and 12 min for 2.4 to 2.7 V, the ratio (0.063) of those two times is expressed as T_0.2 C_. As for photocharge process with active area of 0.42 cm^2^, $T_{0.42\;{\mathrm{cm}}^{2}}$ is 0.69 for the fabricated PSC–Li–S battery (the charge times for 1.7–2.4 and 2.4–2.7 V are 29 and 20 min, respectively). T factors of different photocharge active area, and galvanostatically charged at high rates are shown in Figure 3c, the discharge capacity performance at 0.2 C for different charge condition is presented as well. Although T values are gradually increased from 0.2 to 4 C under the power supply mode, the capacity of Li–S battery quickly decreases from 752.8 to 420.4 mAh g^−1^, showing poor charge efficiency at high rates. For the fabricated PSC–Li–S battery, the photocharge effect with high T factors is positive as compared to power supply mode. In particular, the discharge capacity could be retained at around 750 mAh g^−1^, clarifying good charge efficiency of the solar driven Li–S battery based on the self‐adjusting charge feature, like dual‐mode charge process in Li‐ion batteries.

To measure the stability of the fabricated PSC–Li–S battery, cyclic tests are conducted (**Figure** **4**a). Obviously, the capacity remains at around 750 mAh g^−1^ after 14 cycles (the testing time is more than 50 h, which is significantly longer than previously reported works) with almost the similar photocharge/discharge profiles, indicating the stable capacity performance, and voltage polarization. It should be mentioned that the devices can work with the stable capacity for even more cycles. Nevertheless, the photocharge time gets longer with increasing the cycle number due to the slow decrease of PCE of PSCs during cycling. Although the device can retain the good performance under light or thermal treatment within 120 min (Figures S2, S3, and S7, Supporting Information) after being capsulated carefully and firmly (Figure S6, Supporting Information). However, the long‐term stability of the device is still limited. During the rather long working process (more than 3000 min), the cross impact of light and heat would induce degradation of PSCs. Meanwhile, the inevitable leakage and diffusion of the electrolyte would also be harmful for the device. In this work, the test is ended when the photocharge time is doubled that in the first cycle.

**Figure 4:**
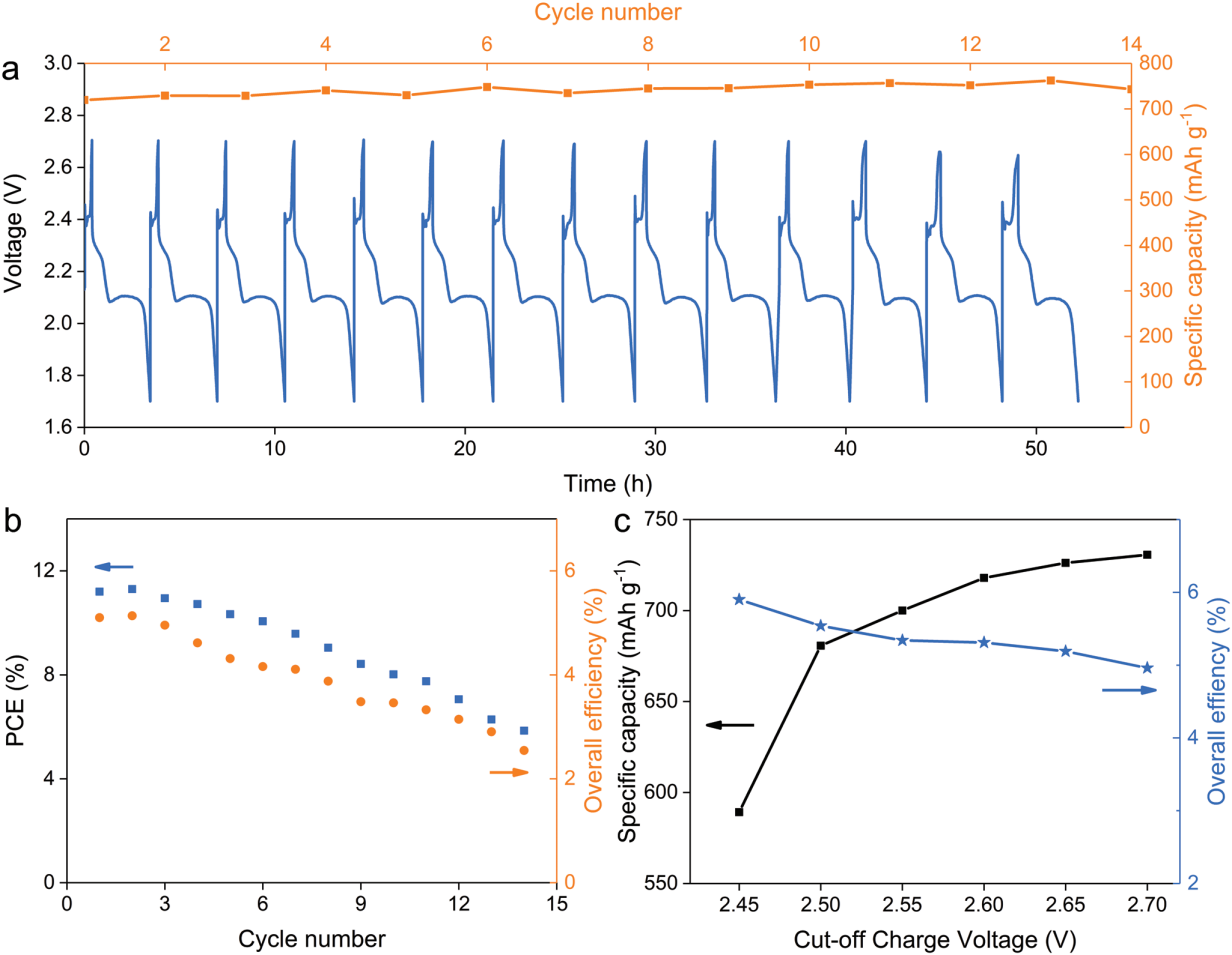
a) Cyclic discharge capacity performance (yellow) and voltage–time profiles (blue) of the fabricated PSC–Li–S battery in photocharge process with an active area of 0.63 cm^2^ on PSCs part. b) PCE and overall efficiency as a function of cycle number. c) Discharge capacity and overall efficiency of different cut‐off photocharge voltage.

The energy conversion efficiency is the primary concern for all photovoltaic technologies. Herein, the overall efficiency is an important parameter, which is usually lower than 1% for solar storable systems based on DSSC. In general, the energy conversion, storage and utilization of the solar rechargeable battery can be achieved via three stages: solar to electrical energy conversion, electrical‐to‐chemical energy conversion simultaneously in the photocharge process, and chemical‐to‐electrical energy conversion subsequently in the discharge process. Briefly, the overall energy conversion efficiency (η_overall_) of the solar rechargeable battery mainly depends on PCE and electrical‐to‐chemical conversion efficiency (η_EC_) of the PSCs part to photocharge the Li–S battery part under irradiation, and chemical‐to‐electrical conversion efficiency (η_CE_) by the electrochemical way in the dark. Since there are still other power losses, the precise overall efficiency is calculated as the ratio of discharge energy and illumination energy for particular active areas. All the efficiencies are calculated according to step 1 and 2, and results are shown in Figure 4b. Obviously, the high overall efficiency of 5.14% can be obtained in the first cycle for the fabricated PSC–Li–S battery. During cycling, the overall efficiency has the same decay trend with PCE, indicating that the overall efficiency of the solar rechargeable battery highly relies on the photovoltaics side. Although the PSCs part presents a steady output in a short‐time steady output test (Figure 2e), the total irradiate time would exceed 500 min in air atmosphere during cyclic photocharge process. Under this operation circumstance, the efficiency of PSCs would decrease from about 11.24% to 5.85% after 14 cycles. At the same time, the overall efficiency of the solar rechargeable battery is dropped accordingly from 5.14% to 2.52%. Even in the 14th cycle, the desirable overall efficiency exceeding 2.5% is still superior to most reported works. During this work, we have tried to use mixed‐cation perovskite to improve the performance, but it seems to be inefficient and unstable in our system when combined with carbon electrode. Therefore, the typical MAPbI_3_ is chosen in order to remain a relatively stable performance of PSCs. Further optimized works are still needed to improve the stability and efficiency of the integrated system, especially the PSCs part according to our discussion above. Highly stable and effective perovskite components are preferred, interfacial passivation and surface protection technologies would also be helpful.^52^, ^53^ Using solid‐state electrolyte to avoid the leakage could be an alternative solution, However, the low conductivity and large interface impedance of solid‐state electrolyte are under the improvement.^54^ If those obstacles can be overcome, the fabricated PSC–Li–S battery would achieve more attractive long‐time working performance. Meanwhile, the charge time and cut‐off voltage in photocharge process are also important to the overall efficiency as discussed before. Taking into account of the large discharge capacity (580 to 730 mAh g^−1^), the overall efficiency can be optimized from 5.9% to 4.96% in cut‐off voltage from 2.55 to 2.7 V (Figure 4c).

In summary, herein we propose an integrated PSCs/Li–S solar driven rechargeable battery based on a new mode of JEM for the first time. The carbon‐based PSCs pack shows an excellent PCE of 12% (15.9% for the single one) and open‐circuit voltage of 2.8 V, which is high enough to photocharge a high energy Li–S battery. By using a stable joint carbon electrode, Li–S battery with lithium metal anode and sulfur‐loaded carbon cathode is directly assembled above the PSCs part. The compact structure leads to a high overall efficiency of 5.14% from solar to electrical/chemical/electrical energy conversion in the integrated solar rechargeable battery. Owing to the smart self‐adjusting photocharge feature, the Li–S battery can retain high specific capacity up to 750 mAh g^−1^ under ultrafast photocharge rate of 2 C, which would be only 535 mAh g^−1^ at power supply mode under the same condition. These outstanding performance indicate that the JEM‐based solar storable battery developed in this work may open a new gate for high energy and effective solar storable systems with new charge mode.

## Experimental Section

*Materials Preparation*: Etched FTO and CH_3_NH_3_PbI_3_(MAI) were purchased from YOUXUAN Tech (Yingkou, China), PbI_2_ and CsI were purchased from p‐OLED Corporation (Xi'an, China). Commercial carbon paste was purchased from Shanghai MaterWin New Materials Corporation (China). Sulfur element and carbon nanotubes were purchased from J&K chemical Tech (China).

*PSCs–Sulfur and Carbon–Lithium Devices Assemble*: Etched FTO glasses were cleaned sequentially with detergent, deionized water, alcohol, and acetone, followed by drying with N_2_ flow and O_2_ plasma treatment for 5 min. The bis(pentane‐2,4‐dionato‐O,O′)bis(propan‐2‐olato) titanium solution (J&K chemical Tech, China) was spin‐cast onto FTO electrodes at 5000 rpm for 40 s. The film was annealed at 120 °C for 10 min and 500 °C for 1 h. The FTO/TiO_2_ substrate was then transferred to an Ar‐filled glove box. The perovskite layer was deposited on the TiO_2_ layer by a spin‐coating method. In detail, 461 mg PbI_2_, 151 mg MAI, 13 mg CsI, and 78 mg dimethylsulfoxide were mixed in 1 mL *N*,*N*‐dimethylformamide. Perovskite precursor solution (50 µL) was dropped on the substrate and spin‐coated at 1000 rpm for 10 s and 4000 rpm for 20 s with chlorobenzene (100 µL) being dripped on the substrates 14 s prior to the end of the process. The substrates were then annealed at 120 °C for 10 min to obtain crystalline perovskite films. After cooling down, carbon paste was coated on the top of perovskite by doctor‐blading method, followed by 120 °C heated for 15 min. Sulfur, carbon nanotubes, and polyvinylidene fluoride were blended at a weight ratio of 70:20:10. *N*‐methyl‐2‐pyrrolidone was used as the solvent. The slurries were stirred in a sealed glass bottle for 4 h and then coated on a piece of carbon paper. After 12 h in a 60 °C dry oven, the carbon paper was paste on the top of the carbon electrode of PSCs and heated at 100 °C for 5 min to get a good contact, a glass should be coated to reduce the sulfur loss. The S mass loading was controlled at 0.85–1 mg cm^−2^, the active area was determined as 0.78 cm^2^. Li foils and Celgard 2300 film acted as the counter electrode and separator, the electrolyte was composed of 1 m bis(trifluoromethane) sulphonamide lithium salt (LiTFSI) dissolved in a mixture of 1,3‐dioxolane and dimethoxymethane (1:1 by volume) with 0.1 m LiNO_3_ additive. The integrated devices were fabricated in Ar‐filled glove box, and compressed by a Hoffmann clip. The seal process was achieved by using ethylene–vinyl aetate copolymer film and further encapsulated by a hot glue gun.

*Measurements*: All the devices were tested in air condition using a Keithley 2400 source meter and a Newport Oriel sol2A solar simulator (300 W). The 91150 V Reference Cell and Meter (ORIEL instrument) was used to calibrate the light intensity to be 100 mW cm^−2^ before the device testing. The device performance parameters were obtained from the current–voltage curves of the solar cells under illumination.

*Calculations*: 1. The energy‐conversion efficiency of the PSC (PCE)

(1) $$\mathrm{PCE}=J_{\mathrm{sc}}\;*V_{\mathrm{oc}}\;*\mathrm{FF/}P\;*\mathrm{100}\%$$

where *J*_sc_, *V*_oc_, FF, and *P* are short‐circuit current density (mA cm^−2^), open‐circuit voltage (V), fill factor, and incident light power density (100 mW cm^−2^), respectively.

2. The overall energy‐conversion efficiency for the entire integrated unit

(2) $$\eta_{\mathrm{overall}}\;=\;E_{\mathrm{discharge}}/\left(P*S*T\right)\mathrm{*100}\%$$

where *E*_discharge_, *P*, *S*, and *t* are discharge energy of Li–S part (mWh, from Land machine), light power density (100 mW cm^−2^), effective area of PSCs in series (cm^2^), and photocharge time (h), respectively.

## Conflict of Interest

The authors declare no conflict of interest.

## Supporting information

Supplementary
