# Supplementary material for: Solar‐Driven Rechargeable Lithium–Sulfur Battery
Source: Adv Sci (Weinh). 2019 May 24;6(15):1900620. doi: 10.1002/advs.201900620 (PMC6685504; doi:10.1002/advs.201900620)
Supplement: Supplementary file 1 — Supplementary [file ADVS-6-1900620-s001.pdf]

Copyright WILEY-VCH Verlag GmbH & Co. KGaA, 69469 Weinheim, Germany, 2019.

ADVANCED SCIENCE
Open Access

Supporting Information

for *Adv. Sci.,* DOI: 10.1002/advs.201900620

Solar-Driven Rechargeable Lithium–Sulfur Battery

*Peng Chen, Guo-Ran Li, Tian-Tian Li, and Xue-Ping Gao**

Supporting Information

Solar-Driven Rechargeable Lithium-Sulfur Battery

*Peng Chen, Guo-Ran Li, Tian-Tian Li, Xue-Ping Gao**

Institute of New Energy Material Chemistry, School of Materials Science and Engineering, Renewable Energy Conversion and Storage Center, Nankai University, Tianjin 300350, China, E-mail: xpgao@nankai.edu.cn

Discussion and details about T factor:

For a typical charge-discharge process in Li-S battery, the reaction could be written as following[1]:

$$16Li+S_8 \rightarrow 16Li^{+}+8S^{2-}$$

$$E = E^{\theta} - \frac{RT}{nF} \ln \frac{(a_{Li^+})^{16}(a_{S^{2-}})^{8}}{a_{Li}a_{S_8}}$$

Where E, E^{θ}, R, T, n, F, a are electrode potential, standard electrode potential, ideal gas constant, Kelvin temperature, electron transfer number, Faraday constant and activity of component, respectively.

Obviously, the rapid change of ion concentration nearby the electrode would cause drastic fluctuation of the apparent potential. For Li-S battery, the power supply charger (galvanostatical charging process) would cut off the charge process whenever the apparent voltage of Li-S battery reaches 2.7 V, despite of the actual reaction extent. In photo-charge process of the solar rechargeable battery (**Figure 3a**), when the voltage increases from 2.4 V to 2.7 V, the current decreases simultaneously. It means that the charging current and voltage are limited by PSCs part and decrease rapidly (**Figure 2b, 2d**), unlike galvanostatically charging. In this way, the current would be auto-adjusted with the change of ionic concentration nearby the electrode during photo-charge process. As a result, the charge time at high-voltage range is prolonged, the ratio of charge time at high-voltage and low-voltage range (T factor) would increase accordingly.

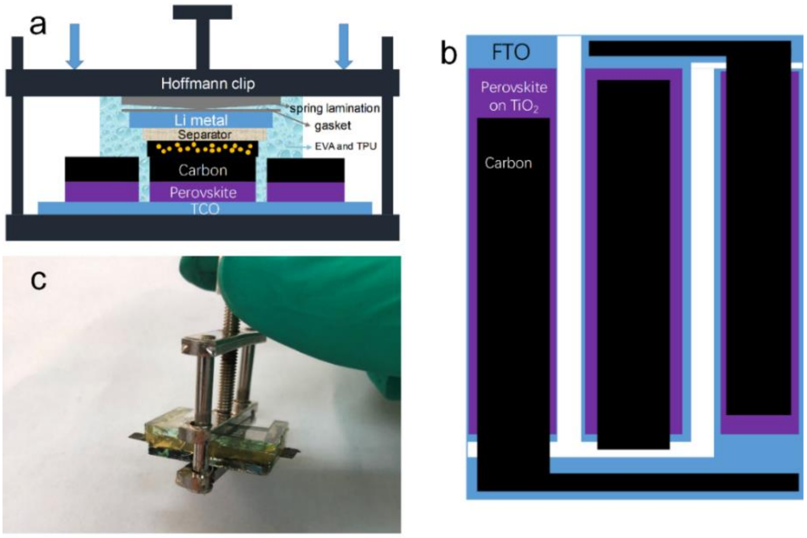

Figure S1. (a) Schematic of the integrated device. (b) Schematic of the serial connected PSCs (c) Digital image of integrated device.

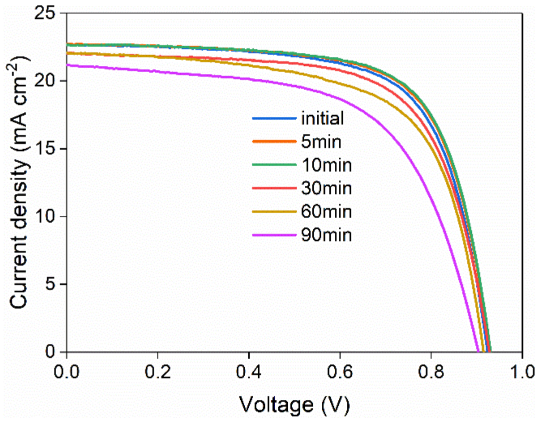

Figure S2. *J*-*V* curves under continuous irradiation for 90 min.

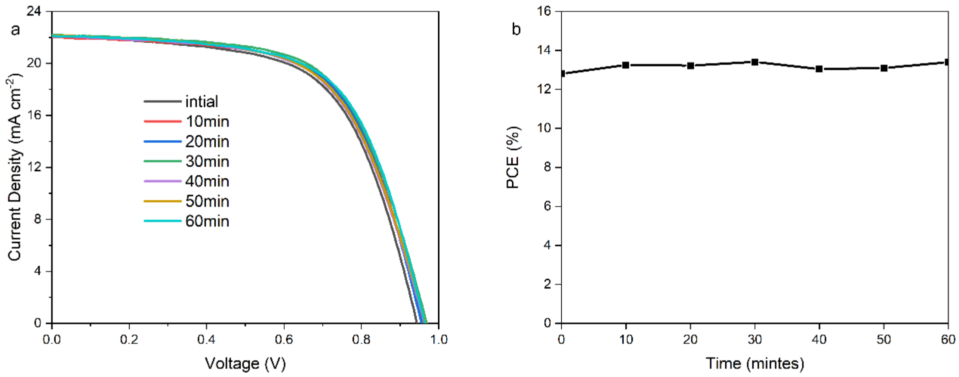

Figure S3. (a) J-V curves for perovskite solar cells with different heat treatment time at 100℃. (b) PCE change of perovskite solar cells with different heat treatment time at 100℃.

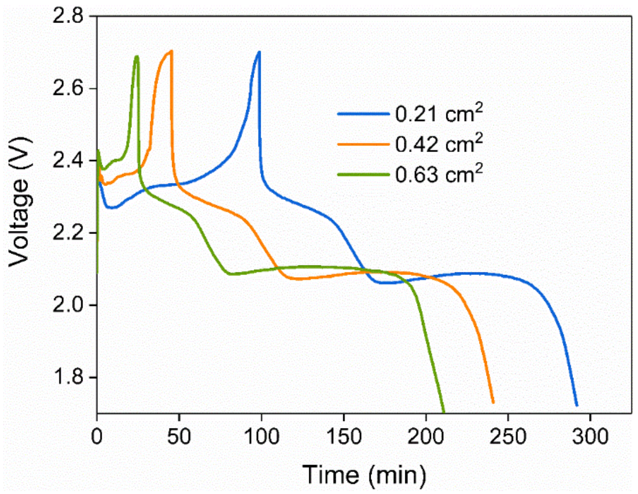

Figure S4. Voltage-time curves for different active areas for photo-charge mode.

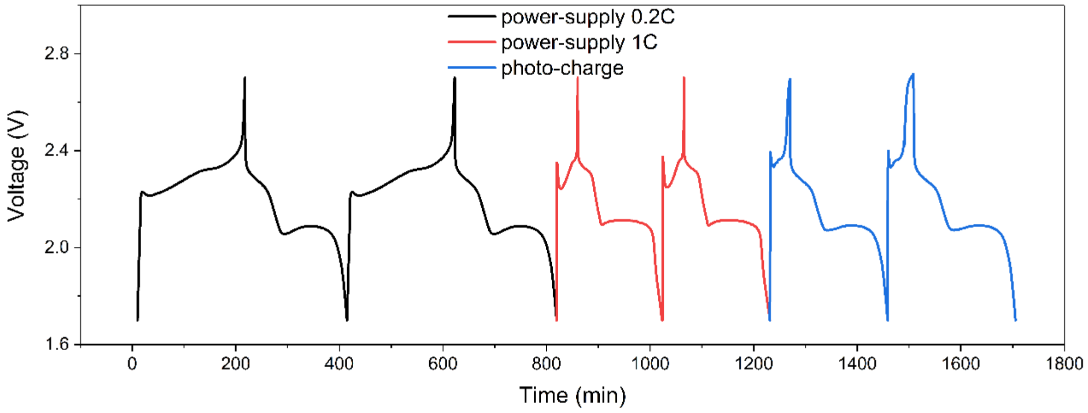

Figure S5. Voltage-time curves of 0.2C and 1C for power supply mode versus photo-charge mode. The charge time is similar for 1C and photo-charge mode.

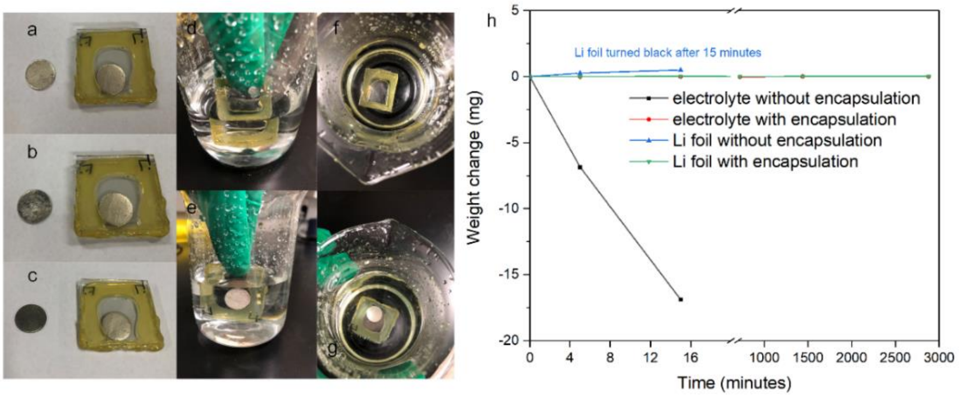

Figure S6. (a-c) Li foil with and without encapsulated been exposed in air for different time. (d-g) Digital images of sealed electrolyte and Li foil dipped in water. (h) Weight change of four samples versus time.

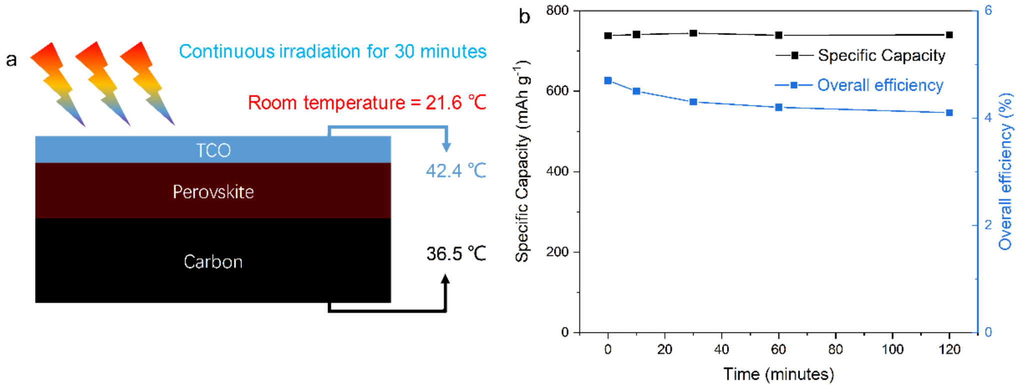

Figure S7. (a) Temperature distribution of the integrated device under irradiation. (b) Performance of integrated device aged for different time at 50 ℃.

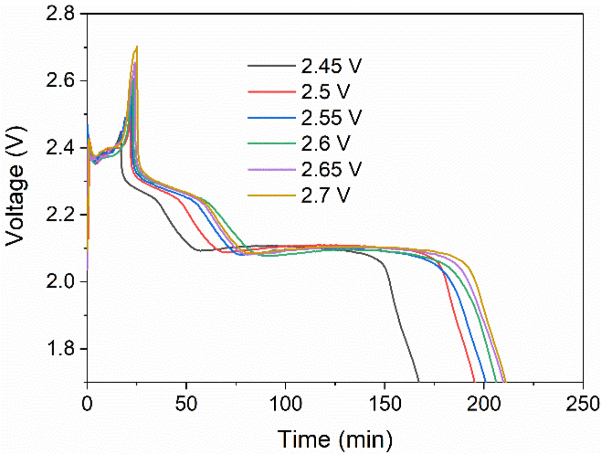

Figure S8. Voltage-time curves for different cut-off voltage.

Reference

[1] Y. V. Mikhaylik, J. R. Akridge, *Journal of The Electrochemical Society* **2004**, *151*, A1969-A1976.
